# Computational Analysis of Chemical Space of Natural Compounds Interacting with Sulfotransferases

**DOI:** 10.3390/molecules26216360

**Published:** 2021-10-21

**Authors:** Iglika Lessigiarska, Yunhui Peng, Ivanka Tsakovska, Petko Alov, Nathalie Lagarde, Dessislava Jereva, Bruno O. Villoutreix, Arnaud B. Nicot, Ilza Pajeva, Tania Pencheva, Maria A. Miteva

**Affiliations:** 1 Department of QSAR and Molecular Modelling, Institute of Biophysics and Biomedical Engineering, Bulgarian Academy of Sciences, 1113 Sofia, Bulgaria; iglika@biomed.bas.bg (I.L.); itsakovska@biomed.bas.bg (I.T.); petko.alov@biophys.bas.bg (P.A.); dessislava.jereva@biomed.bas.bg (D.J.); pajeva@biomed.bas.bg (I.P.); 2 INSERM U1268 “Medicinal Chemistry and Translational Research”, CiTCoM UMR 8038 CNRS—Université de Paris, 75006 Paris, France; yunhuip@g.clemson.edu; 3 Department of Physics and Astronomy, Clemson University, Clemson, SC 29634, USA; 4 Laboratoire GBCM, EA7528, Conservatoire National des Arts et Métiers, 2 Rue Conté, Hésam Université, 75003 Paris, France; nathalie.lagarde@lecnam.net; 5 INSERM UMR 1141, Robert-Debré Hospital, 75019 Paris, France; bruno.villoutreix@inserm.fr; 6 INSERM, Nantes Université, Center for Research in Transplantation and Translational Immunology, UMR 1064, ITUN, F-44000 Nantes, France; arnaud.nicot@inserm.fr

**Keywords:** sulfotransferase, SULT1A1, natural compounds, ANOVA, PCA, cluster analysis, docking, chemical space, polyphenol, flavonoid

## Abstract

The aim of this study was to investigate the chemical space and interactions of natural compounds with sulfotransferases (SULTs) using ligand- and structure-based in silico methods. An in-house library of natural ligands (hormones, neurotransmitters, plant-derived compounds and their metabolites) reported to interact with SULTs was created. Their chemical structures and properties were compared to those of compounds of non-natural (synthetic) origin, known to interact with SULTs. The natural ligands interacting with SULTs were further compared to other natural products for which interactions with SULTs were not known. Various descriptors of the molecular structures were calculated and analyzed. Statistical methods (ANOVA, PCA, and clustering) were used to explore the chemical space of the studied compounds. Similarity search between the compounds in the different groups was performed with the ROCS software. The interactions with SULTs were additionally analyzed by docking into different experimental and modeled conformations of SULT1A1. Natural products with potentially strong interactions with SULTs were outlined. Our results contribute to a better understanding of chemical space and interactions of natural compounds with SULT enzymes and help to outline new potential ligands of these enzymes.

## 1. Introduction

Drug-metabolizing enzymes are involved in the metabolism of endogenous molecules and the elimination of xenobiotics and drugs [1,2,3,4]. Phase I metabolism includes oxidation and reduction reactions, while Phase II comprises conjugation reactions [5]. Cytosolic sulfotransferases (SULTs) participate in Phase II reactions in the body [6] by catalyzing the sulfuryl group transfer from the co-factor 3′-phosphoadenosine 5′-phosphosulfate (PAPS) to a substrate hydroxyl or amino group of endogenous/exogenous compounds [7,8,9,10]. At high concentrations, some substrates inhibit SULTs and dead-end complexes with bound inactive co-factor PAP can be created. The cytosolic SULTs include 13 enzymes that can be found in many tissues [11]. SULTs metabolize a wide variety of substrates, including endogenous compounds like steroids and polysaccharides, natural compounds [12,13] and drugs [8]. The molecular mechanisms involved in the substrate specificity of different SULT isoforms have been previously studied [14,15,16,17,18,19,20,21,22,23,24,25].

In this study, we investigated human SULT1A1 [26], which is the most abundant SULT in human liver, and is also distributed in lung, platelets, kidney, and gastrointestinal tissues [27]. Human SULT1A1 exhibits a broad substrate range with specificity for phenolic compounds, including drugs and pro-carcinogens such as N-hydroxy-aromatic and heterocyclic arylamines [8]; indeed, the SULTs play a key role in the metabolism of a number of natural compounds [12,28,29,30,31]. Contrary to many exogenous compounds, some natural compounds (e.g., flavonolignans) are sufficiently functionalized by Phase II conjugations without the need to pass through Phase I metabolism [12]. In human hepatocytes, flavonolignans were found to be metabolized by glucuronidation or sulfation, while other types of conjugations, such as methylation or glutathionylation, or formation of Phase I products, were negligible [32,33]; the flavonoid taxifolin can also be metabolized directly by Phase II conjugations [34,35].

Here, we focused on the chemical space and interactions of natural compounds with SULTs using ligand- and structure-based in silico methods. We created an in-house library of natural ligands reported to interact with SULTs, and the chemical structure and properties of the compounds were compared to the properties of synthetic compounds known to interact with SULTs. The natural ligands interacting with SULTs were further compared to other natural products for which interactions with SULTs were not known. Cluster and similarity analyses were performed between the compounds, and their interactions with SULTs were analyzed by docking into experimental and modeled conformations of SULT1A1. Natural products with potentially strong interactions with SULTs were proposed, thus outlining them as potential SULT ligands.

## 2. Results and Discussion

The groups of the studied compounds are presented in Table 1.

The descriptors of the molecular structures available in Molecular Operating Environment (MOE), v.2019.0102 (Chemical Computing Group, https://www.chemcomp.com/) were calculated and analyzed. Differences in the descriptor means among the three groups of compounds were evaluated with ANOVA. Principal component analysis (PCA) was used to extract information from the calculated descriptors; cluster analysis was applied to the chemical compounds. ROCS similarity search between the compound groups was performed.

The interactions of the three groups of compounds with SULTs were further analyzed by docking to three alloforms of SULT1A1, the crystallographic structures of which were retrieved from the Protein Data Bank (PDB, https://www.rcsb.org). 

### 2.1. Natural SULT Ligands

#### 2.1.1. Compounds Collection for the In-House Library of Natural SULT Ligands

The search in the scientific literature [15,36,37,38,39,40,41,42,43,44,45,46,47,48,49,50,51,52,53,54,55,56,57,58,59,60,61,62,63,64] for natural compounds reported as ligands of SULTs led to the creation of an in-house library of 118 structures. It is freely available at http://biomed.bas.bg/qsarmm/.

Sixty-two compounds were reported as substrates, 53 compounds were inhibitors of SULT1A1, and for 3 compounds, both substrate and inhibitory kinetic parameters were available. Most of the compounds were also reported to be substrates or inhibitors of other SULT isoforms, as listed in the database. 

The library of collected natural SULT ligands contains the following information:

- SMILES and InChi keys structure notations;
- the trivial name of the ligand as provided in the literature;
- PubChem CID;
- ligand type (substrate/inhibitor);
- binding affinity data (IC50, Km), where available;
- the SULT isoform the ligand interacts to;
- physico-chemical parameters important for ADME properties: molecular weight, n-octanol/water partition coefficient, aqueous solubility, number of hydrogen atom donors/acceptors, calculated in MOE;
- literature sources.

#### 2.1.2. Characterization of the Chemical Space of the Natural SULT Ligands

*Basic statistics of the calculated structural descriptors*

In the present research, 322 structural and physico-chemical descriptors of the 118 natural SULT ligands were calculated (see Section 3). After removing the descriptors without variance, 313 chemical descriptors remained for the analyses. The number of Br, Cl, and F atoms had zero values for all compounds.

The following molecular descriptors commonly used for characterizing compounds’ pharmacokinetic behavior and interactions between the three investigated groups were analyzed and compared:

- ASA+—water accessible surface area of all atoms with positive partial charge;
- ASA−—water accessible surface area of all atoms with negative partial charge;
- ASA_H—water accessible surface area of all hydrophobic atoms;
- ASA_P—water accessible surface area of all polar atoms;
- n_acc—number of H-bond acceptor atoms;
- n_don—number of H-bond donor atoms;
- rings—number of rings;
- volume—van der Waals volume;
- surface—van der Waals surface area;
- weight—molecular weight;
- logP(o/w)—octanol-water partition coefficient.

The statistics of the above listed chemical descriptors in the group of the investigated natural SULT1A1 ligands are reported in Table 2.

Most of the natural SULT ligands are of medium size (average MW of 272 Da) and contain at least one ring (3 rings on average), but there are also some large molecules with a high number of rings (up to 11). On average, the number of H-bond donor or acceptor atoms is 3 and 4, respectively, but some ligands do not have H-bond donors (flavone, bergamottin, nobiletin, rotenone, and tangeretin) or have only one H-bond acceptor, while others have as much as 17 H-bond donors and 24 H-bond acceptors (punicalagin). The ASA+ of the molecules is slightly larger than the ASA−. The hydrophobic/hydrophilic properties vary from very hydrophilic molecules with negative logP(o/w), reaching −2.1, to hydrophobic with high positive logP(o/w) (up to 5.3); the average logP(o/w) is 2.2. In general, there are more hydrophobic molecules with positive logP(o/w) and larger hydrophobic surface areas than hydrophilic molecules. The observed diversity in the structural characteristics of the natural SULT ligands in relation to the size, H-bonding properties, and hydrophobic/hydrophilic properties is in agreement with the well-known promiscuity of SULTs but with preferential selectivity towards a specific class of compounds for each isoenzyme [6].

*Principal Component Analysis of the calculated structural descriptors*

**Figure 1:**
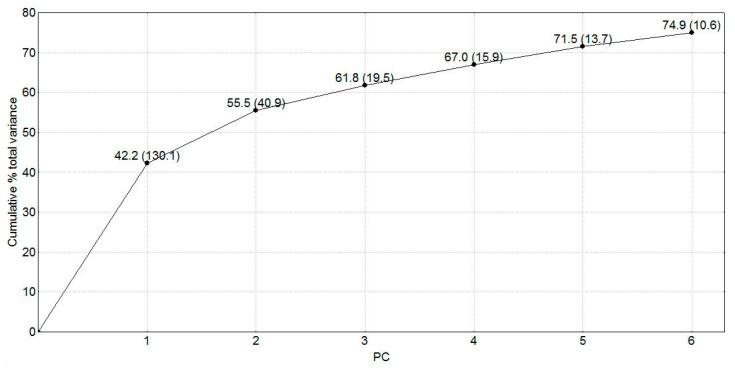
Plot of percentage of the explained variance of the descriptors by the first six principal components (PC). The PC eigenvalues are given in brackets.

PCA was applied in order to analyze the set of 313 calculated descriptors for natural SULT ligands. The eigenvalues of the principal components (PC) and the percentage of the explained variance of the descriptor data set are presented in Figure 1:

The first two components covered 55.5% of the descriptor variance. The analysis of the projections of the original variables onto the component plane (PCA loadings) revealed that the first PC (PC1) accounted mostly for the steric characteristics/size (molecular weight, volume, surface, and shape), while logP(o/w), ASA_H, ASA_P, and hydrophobic volume contributed to the second PC (PC2), thus accounting for the hydrophobic/hydrophilic properties.

**Figure 2:**
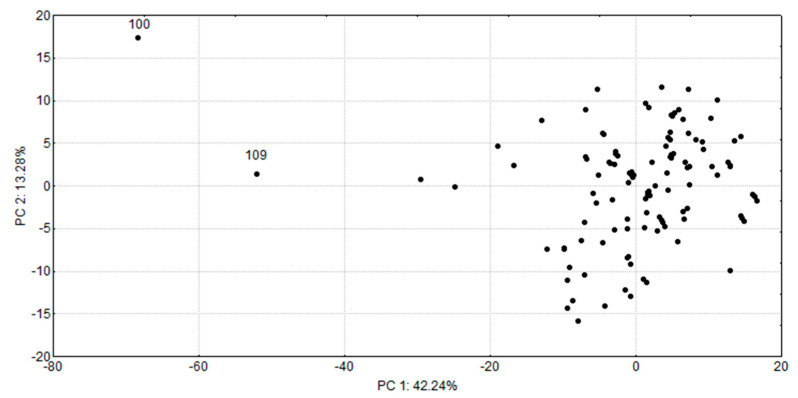
PCA score plot (PC1 vs. PC2) based on the descriptors of the natural SULT ligands.

A plot of projection of the compounds onto the PC plane (the score plot of PC1 vs. PC2) is presented in Figure 2:

Compounds 100 and 109 (punicalagin and thearubigin), which fall outside of the rest compounds, are very bulky and have the highest molecular weights among the natural SULT ligands. No similarity groups could be identified within the investigated compounds, confirming the diversity in their chemical structures. 

*Cluster analysis*

**Figure 3:**
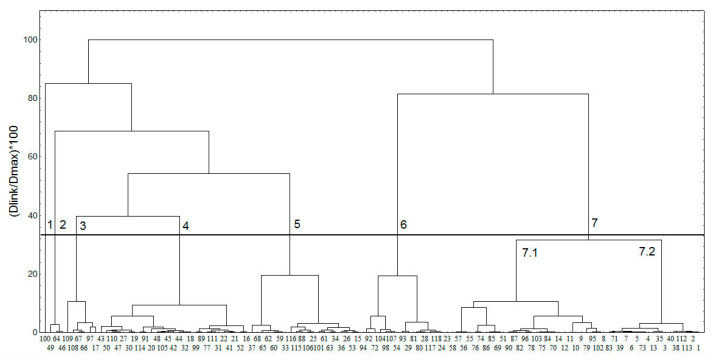
Clusters obtained in the group of natural SULT ligands. For clarity, the compound numbers are presented in two lines: the compounds on the second line are positioned in the cluster between the corresponding compounds on the first line. The 33% cutoff line is shown, and the clusters are numbered.

To better characterize the chemical space of the studied compounds, five principal components, describing 72% of the variance in the compound structures, were utilized in the cluster analysis (Figure 1). The obtained clusters are presented in Figure 3; the clusters were identified by applying a cutoff value of 33% of the maximal distance based on the Sneath’s index of cluster significance (shown as a line in Figure 3) [65]. Seven clusters at the 33% level were outlined. 

**Figure 4:**
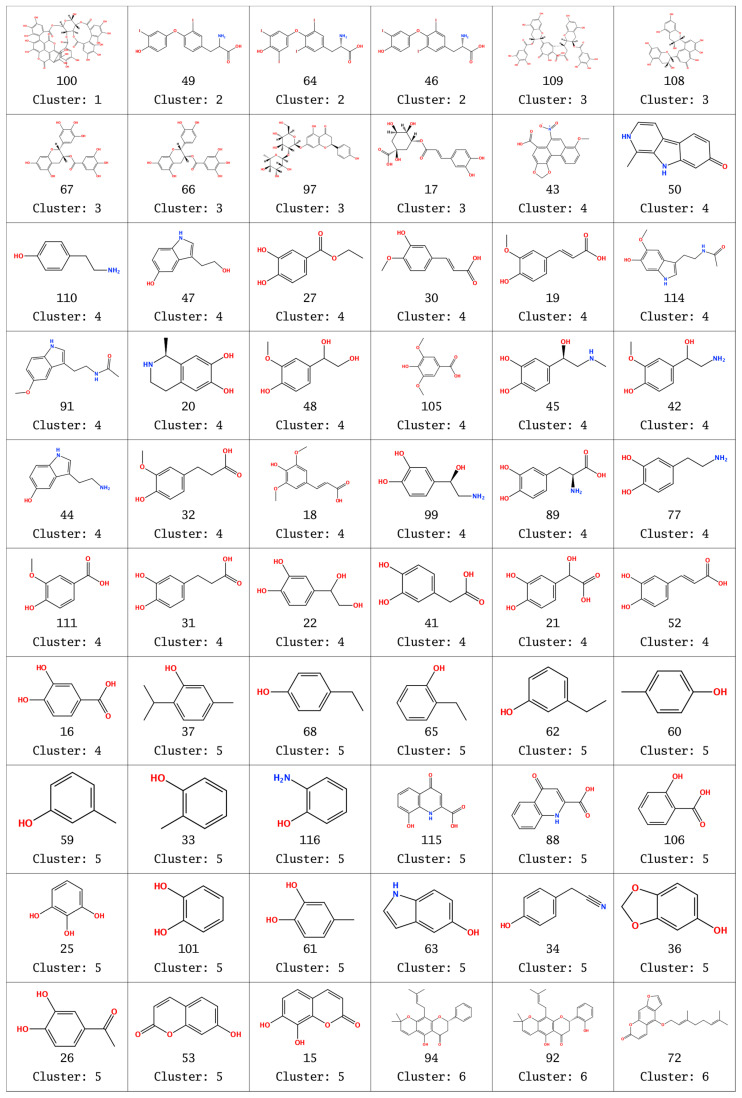

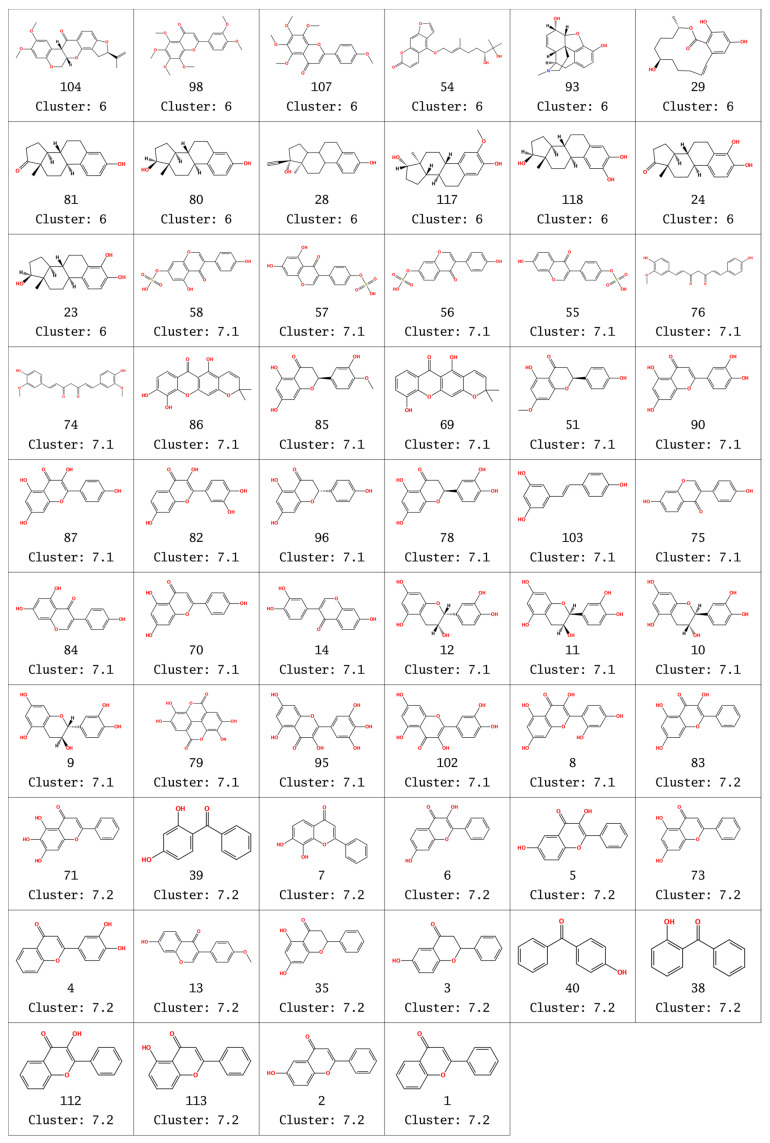
Cluster numbers, compound numbers, and chemical structures of natural SULT ligands.

The chemical structures of the compounds as classified in the clusters are presented in Figure 4.

The first compound classified in a single cluster 1 (100, punicalagin, Figure 3) is very bulky and with the highest molecular weight (1085 Da). As seen in Figure 4, the compounds are reasonably grouped in clusters, according to the molecular descriptors, in agreement with their structures. Cluster 2 includes the three compounds from the group of natural SULT ligands, which contain iodine. Cluster 3 contains mainly bulky 2-phenylchroman (flavan) and other 2-arylchroman derivatives (including 1 flavanone derivative). Overall, clusters 4 and 5 include compounds with one or two fused rings, but cluster 4 has compounds with more oxygen or nitrogen atoms, compared to the compounds in cluster 5. Cluster 6 comprises compounds with more than two rings, including also some steroid compounds—derivatives of estrone and estradiol. Cluster 7 contains mainly hydroxy flavones; there are also some polyphenol compounds, such as curcuminoids, stilbenes, and benzophenones, with similar structural fragments. Cluster 7 may be divided into two smaller clusters (denoted as 7.1 and 7.2), close to the 33% distance level (Figure 3). In general, the compounds in cluster 7.2 contain lower number of oxygen atoms than the compounds in cluster 7.1 and have an unsubstituted phenyl ring.

#### 2.1.3. Docking Results

Next, we performed docking of all 118 compounds into different SULT1A1 conformations, using MOE software. We applied two scoring functions, Alpha HB and London dG with different terms of the binding energies calculations (see Section 3 for details). 

**Figure 5:**
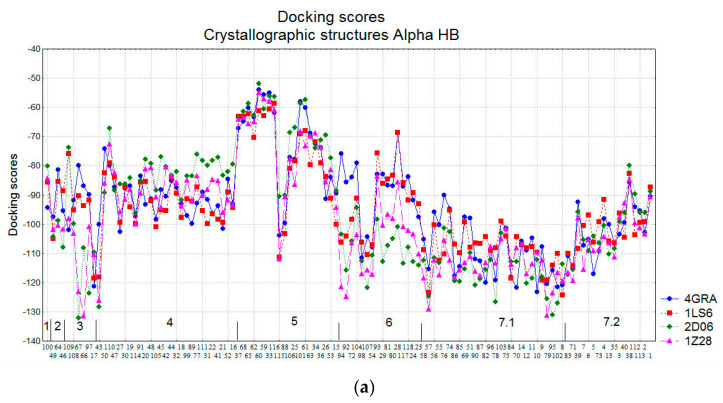

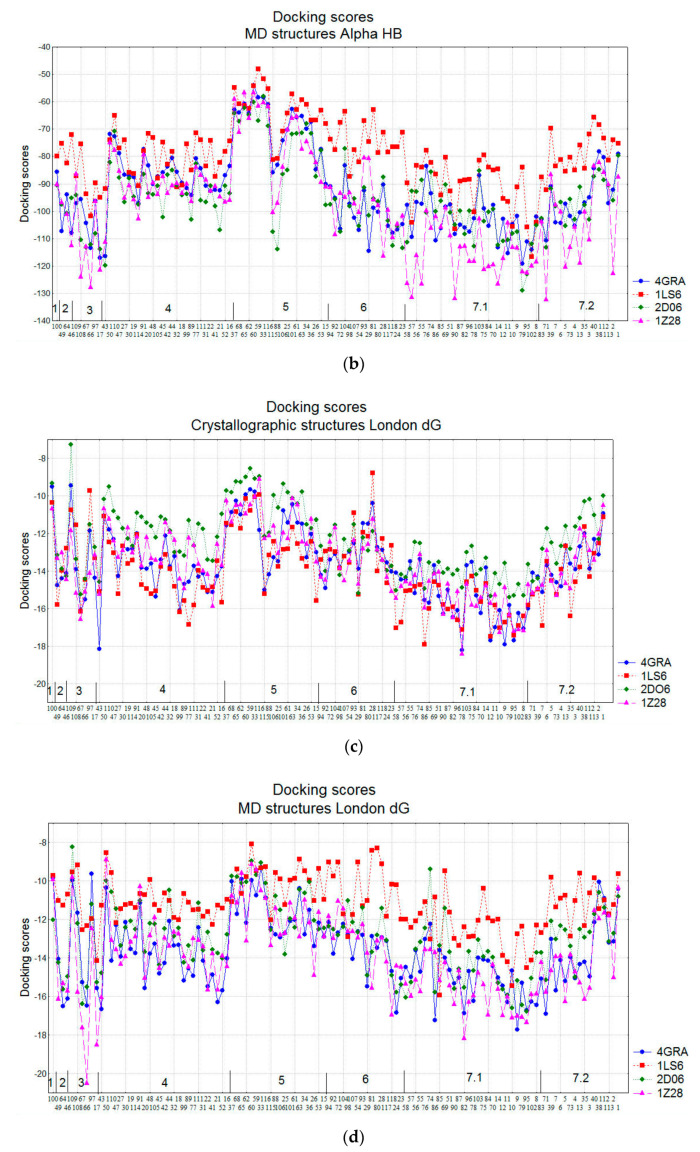
Docking scores for the natural SULT ligands. (**a**) Crystallographic structures of SULT1A1 using Alpha HB scoring function; (**b**) MD generated conformations of SULT1A1 using Alpha HB scoring function; (**c**) Crystallographic structures of SULT1A1 using London dG scoring function; (**d**) MD generated conformations of SULT1A1 using London dG scoring function. The cluster numbers are presented, and the compound numbers are listed in two lines: the compounds on the second line are positioned in the cluster between the corresponding compounds on the first line.

Figure 5 illustrates the docking scores (approximating the binding energies) obtained for the natural SULT ligands from their docking in eight SULT1A1 structures: four crystallographic (with PDB IDs: 4GRA for SULT1A1*1, 1LS6 and 2D06 for SULT1A1*2, and 1Z28 for SULT1A1*3) and four conformations generated from Molecular Dynamics Simulations (MD) (see Section 3 for details). Alpha HB and London dG scoring functions are applied, and the lowest scores of 30 docking poses for each compound (indicating better binding affinity and stronger interactions with the enzymes) are plotted. The compounds are ordered in the figure, according to the results from the cluster analysis. The cluster numbers are also presented.

The docking scores calculated by different scoring functions could not be directly compared, as they approximate the binding energies, using different terms. Therefore, the comparison between the compounds is based on the trends outlined by the particular scoring values. The docking score range for Alpha HB was between −135 and −50, while for London dG the range was between −21 and −7. Overall, the docking scores followed the same trends for the two scoring functions, and they were similar for the four crystallographic and the 4 MD structures, except for the SULT1A1 conformations generated by the MD simulations starting from the crystal structure 1LS6. The Alpha HB and London dG scores obtained on the crystal structure 1LS6 outperformed those obtained on the corresponding 1LS6 MD structures. Previously, we had obtained similar results in terms of scores of drug-like molecules docked into 1LS6 [14]. In that previous study, MD simulations starting from the 1LS6 crystal structure did not permit significant improvement of the scores and the ranks of those compounds [14]. In fact, the 1LS6 structure corresponds to SULT1A1*2 (R213H), which is known to be less stable [66] among the three alloforms studied here, *1, *2 and *3 (see Section 3 for details), and thus the MD simulations without a bound ligand cannot allow the stabilization of the SULT1A1*2 structure.

The lowest binding energies for both Alpha HB and London dG occurred in compound cluster 3 and sub-cluster 7.1. This is expected, taking into account that these clusters mainly consist of phenol-containing compounds (e.g., flavonoids), which are typical substrates of SULT1A1 (also known as phenol sulfotransferase) [67]. Cluster 7.2 also includes flavonoids, but with a smaller number of OH groups, which could explain the worse docking scores. Interestingly, the docking into the MD conformations allowed for the improvement of the Alpha HB and LondondG docking scores of sub-cluster 7.2, underlying the importance of taking into account the conformational changes of SULTs when exploring interactions with their ligands. Our recent study [25], using MD simulations with excited Normal Modes (MDeNM) [68], demonstrated that the natural flexibility of SULT1A1 provides a large opening of the key loops 1, 2, and 3, thus ensuring the recognition of diverse substrates and inhibitors by SULT1A1, the large inhibitor epigallocatechin gallate, in particular [20].

Compound 43 (in cluster 4, Figure 4) had also low binding energies, possibly due to its high number of oxygen atoms, which favors SULT interactions. Some compounds in cluster 6 also had low binding energies in the crystallographic enzyme structures and when applying Alpha HB scoring function.

For the four cases shown in Figure 5a–d, cluster 5 showed very different docking scores due to the different size of its compounds.

### 2.2. Comparison of the Groups of Natural SULT Ligands (118 Compounds), Synthetic SULT Ligands (102 Compounds) and Other Natural Products (1220 Compounds)

#### 2.2.1. Chemical Space Analysis

**Figure 6:**
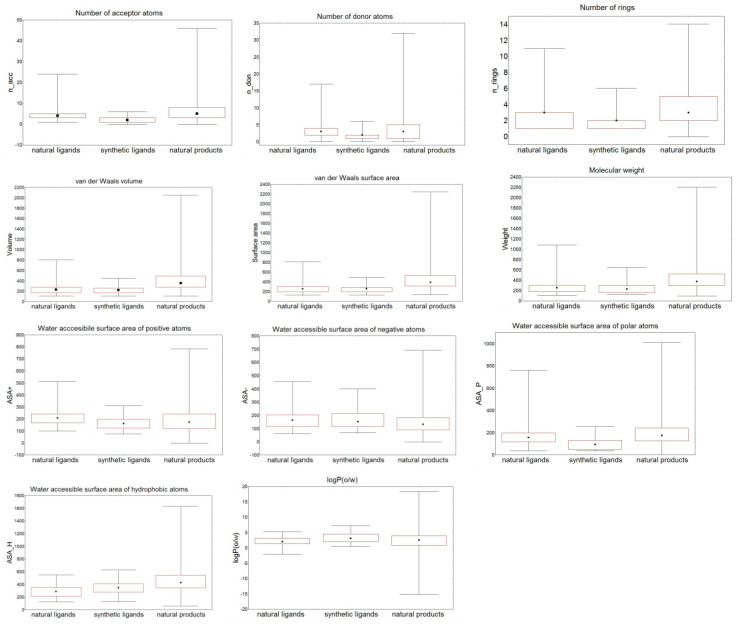
Comparison of the distribution of the selected descriptors in the three compound groups. Median values are presented with dots, 25–75% percentiles are presented with boxes.

In Figure 6, the distribution of several descriptors important for protein-ligand interactions and compound behavior in the living systems is compared in the three studied groups. The minimum–maximum ranges, median values (the dots) and the 25–75% percentiles of the descriptors are presented.

The ranges for most of the descriptors in the synthetic SULT ligands group were narrower and were included in the corresponding descriptor ranges of the natural SULT ligands group. The value ranges of ASA_H and logP(o/w) for the two groups were comparable. The range of logP(o/w) values of the synthetic ligands was slightly higher than the logP(o/w) range of the natural ligands; thus, some synthetic ligands were more lipophilic than the natural ligands.

The range values of the structural descriptors for the natural SULT ligands were within the range of the corresponding descriptor values for the other natural products. For all descriptors, except logP(o/w), the ranges for the natural SULT ligands were in the lowest part of the ranges for the other natural products. The logP(o/w) values for natural SULT ligands were in the middle range of logP(o/w) of the other natural products, showing that there were no extremely hydrophilic or extremely lipophilic natural SULT ligands.

*ANOVA*

ANOVA was used to outline structural descriptors with statistically significant differences between natural and synthetic SULT ligands, and between natural SULT ligands and other natural products. Different descriptor means would suggest different molecular properties between these three compound groups.

Table 3 reports the ANOVA Fisher statistics (F) and the probability values (*p*).

According to the ANOVA results, the H-bond donor and acceptor properties, ASA+, ASA_P, ASA_H, and logP(o/w) are significantly different for the two groups of natural and synthetic SULT ligands at the 99.5% significance level (*p* < 0.005), despite the close descriptor ranges shown above (Figure 6). The molecular size (weight, volume, surface), number of rings, and negatively charged areas are not statistically different in the two groups at the 99.5% significance level, and the compounds may be random samples from the same compound population in relation to these properties.

According to ANOVA, the natural SULT ligands and the other natural products differ in their size and number of rings, H-bond acceptor properties, negatively charged areas, and hydrophobic areas of the molecules. The two groups of compounds have similar means in relation to H-bond donor atoms, logP(o/w), the ASA_P and ASA+. The ANOVA results showed that although the descriptor ranges for natural SULT ligands were within the descriptor ranges for the other natural products, some of the properties for the natural SULT ligands (size, number of rings, H-bond acceptor properties, ASA−, and ASA_H) were significantly different from the other natural products; therefore, a small proportion of natural compound could be SULT ligands. In fact, logP(o/w) of natural ligands interacting with SULTs is within the range of −2.1 to 5.3, which means that such compounds are relatively soluble. However, many of them are with low permeability [29]. The series of metabolic reactions, including Phase I and Phase II reactions, catalyzed by intracellular metabolic enzymes (such as CYP, esterase, SULT, and UGT enzymes) showed that natural medicines with low permeability have distinctive metabolisms and pharmacokinetics [29]. For example, the flavonoid rutin, a natural medicine with antiviral, anti-inflammatory, and vasodilator effects, passes through Phase II metabolism by methylation, sulfation, and glucuronidation reactions [69].

*Cluster analysis*

Natural and synthetic SULT ligands (220 compounds altogether) were combined for the cluster analysis performed further. After removing descriptors without variance in the data set, 311 structural descriptors remained. The PCA extracted six PCs that explained 71.2% of the total variance of the structural descriptors; these PCs were used in the cluster analysis. 

**Figure 7:**
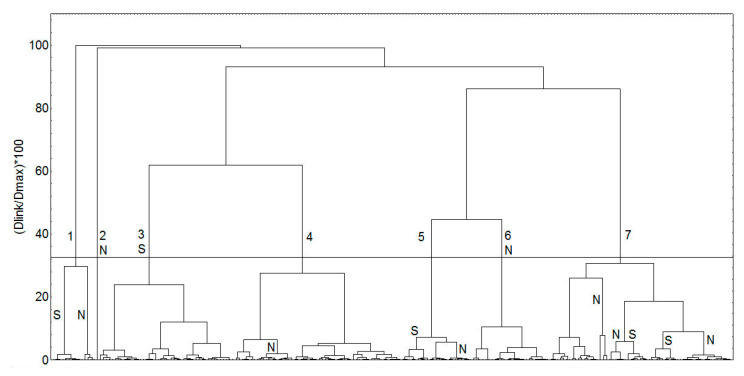
Clusters obtained by combining natural and synthetic SULT ligands. The 33% cutoff line is shown, and the clusters are numbered. Clusters, which contain entirely or predominantly natural or synthetic SULT ligands are labeled with N or S, respectively.

The obtained compound clusters are presented in Figure 7.

Seven clusters at the 33% level were outlined. Some of the clusters contained entirely natural SULT ligands (clusters 2 and 6) or predominantly synthetic SULT ligands (cluster 3); in other clusters, the synthetic SULT ligands were placed together with the natural SULT ligands, although again some sub-clusters of natural or synthetic ligands could be identified, as shown in Figure 7.

Based on the statistical analysis of the structural descriptors, the natural SULT ligands were estimated as slightly more diverse than the synthetic ligands, in agreement with literature reports [70]. The ANOVA analysis showed that the two groups of compounds differ in their means of some molecular properties related to compound polarity (H-bond donor and acceptor properties, ASA+, ASA_P, ASA_H, and logP(o/w)). This was also observed in the cluster analysis, where some natural and synthetic ligands were grouped into different clusters.

**Figure 8:**
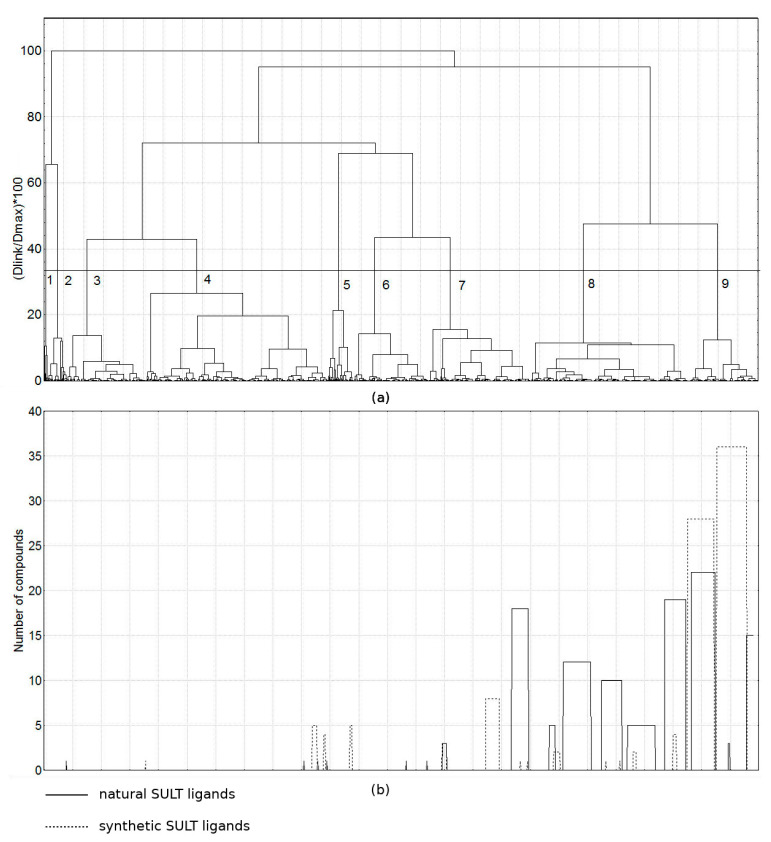
Clusters obtained by combining natural and synthetic SULT ligands and other natural products: (**a**) clusters at the 33% cutoff line are shown and numbered; (**b**) number of natural and synthetic SULT ligands in the clusters presented as columns with height proportional to the number of the SULT ligands in the corresponding cluster.

When combining the three groups—natural SULT ligands, synthetic SULT ligands, and the other natural products (1440 compounds altogether)—318 structural descriptors remained after removing descriptors without variance, and these descriptors were used in the PCA. Seven principal components covered 71.2% of the total variance of the structural descriptors. The obtained compound clusters based on these PCs are presented in Figure 8. The figure also shows the number of natural and synthetic SULT ligands within the clusters by columns.

Nine clusters were outlined. The SULT ligands were distributed among the clusters with the other natural products, suggesting structural similarity, but the ligands were placed mainly in clusters 7, 8 and 9 (these clusters contained 664 compounds). Thus, some structural grouping of the SULT ligands (natural and synthetic) compared to the other natural products was observed. 

*ROCS similarity search*

The chemical similarity between SULT ligands and other natural products was evaluated by the ROCS software. Compounds from the group of other natural products, which are similar to the natural SULT ligands, were sought. We assessed 53 natural compounds as similar to 48 natural SULT ligands, with a Tanimoto-Combo score of >1.5.

#### 2.2.2. Comparison of the Docking Results in the Three Groups of Compounds

**Figure 9:**
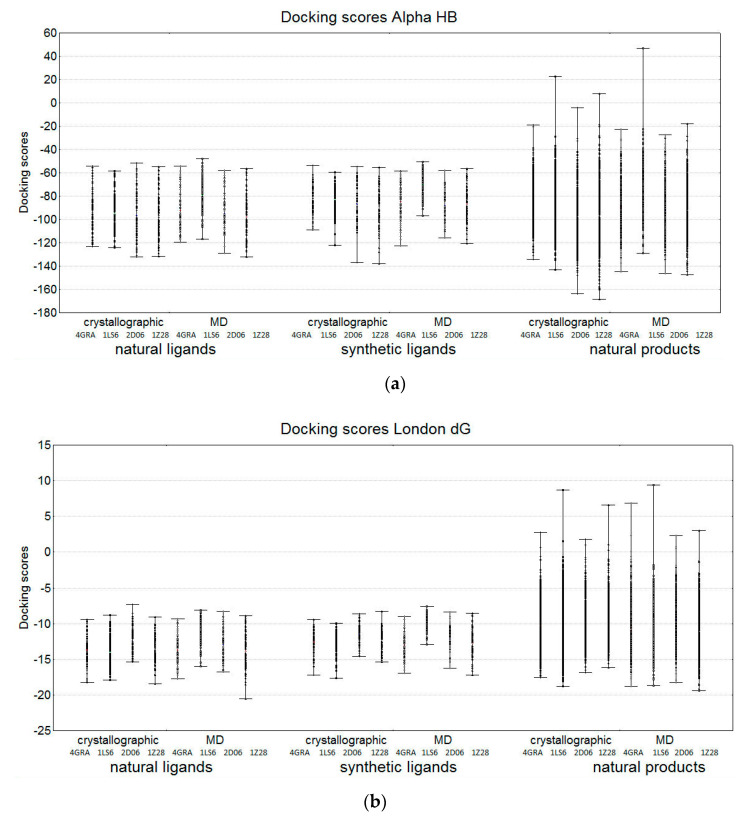
Ranges of the best docking scores obtained by docking the compounds in the crystallographic structures 4GRA, 1LS6, 2D06, and 1Z28, and the corresponding MD structures, using the scoring functions: (**a**) Alpha HB; (**b**) London dG.

The ranges of the best docking scores for each compound in the SULT1A1 structures (four crystallographic structures—GRA, 1LS6, 2D06, and 1Z28—and one MD conformation selected from MD simulations starting from each of the four crystallographic structures), are presented in Figure 9.

As expected, comparable binding energies for the natural SULT ligands and the synthetic SULT ligands were observed, with the natural ligands having a slightly wider range of docking scores. The other natural products had a much broader range of docking scores. The similarity trends obtained for Alpha HB and London dG scoring functions in the group of natural SULT ligands (Figure 5) were also observed in the other groups (Figure 9).

#### 2.2.3. Search for Potential SULT Ligands among the Group of other Natural Products

Further, natural products similar to the natural SULT ligands and natural products showing the lowest binding energies were investigated in order to search for potential active SULT ligands. 

For this purpose, we selected the natural products with an ROCS similarity above 1.5 Tanimoto-Combo score to natural SULT ligands and the natural products showing lower docking energy than the minimal energy obtained for the natural SULT ligands in at least one SULT1A1 structure (crystallographic or MD).

**Figure 10:**
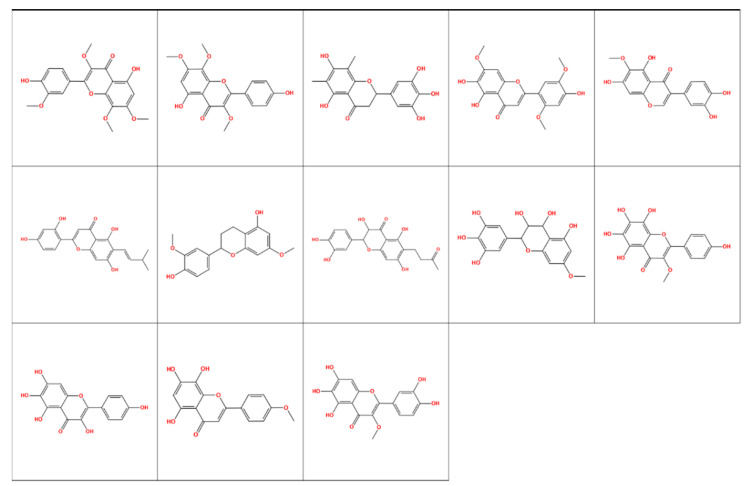
Chemical structures of natural products with high ROCS similarity and lower binding energy compared to natural SULT ligands.

This selection resulted in a group of 159 compounds, where 40 compounds had a ROCS similarity above 1.5 to natural SULT ligands, 106 compounds had lower binding energy than the minimal energy obtained for the natural SULT ligands, and 13 compounds fulfilled both criteria. All these 13 compounds were derivatives of flavone or isoflavone. Their structures are presented in Figure 10.

**Figure 11:**
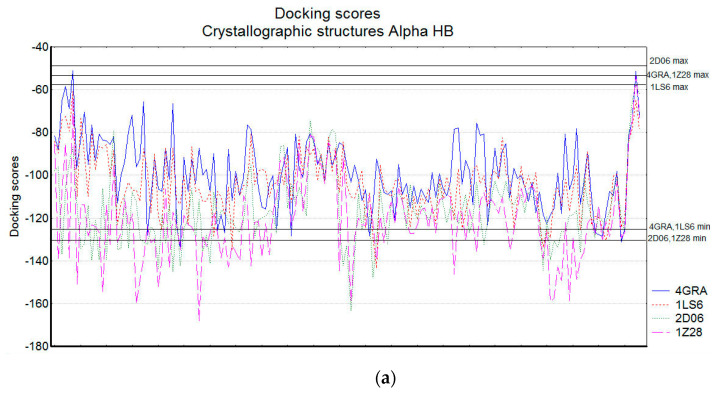

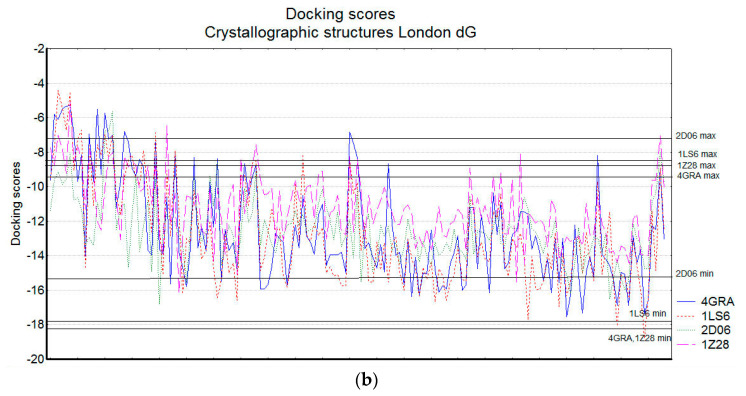
Docking scores of the selected natural products (159 compounds) in the crystallographic enzyme structures using: (**a**) Alpha HB scoring function; (**b**) London dG scoring function. The lines represent the levels of the minimal and maximal scores obtained for the group of natural SULT ligands.

The docking scores of the selected natural products in the crystallographic enzyme structures are presented in Figure 11. The levels of the minimal and maximal scores obtained for the group of natural SULT ligands are shown with lines.

Generally, the docking scores of the selected natural products were located in the lower part of the score ranges for the natural SULT ligands (with some exceptions). Some of the compounds were again flavonoids, others had 3 or 4 fused aromatic rings or 2 phenyl rings with a bridge between them, similar to the compounds in cluster 6 of the natural SULT ligands, which also showed favorable docking scores. These compounds may have strong interactions with SULT1A1.

## 3. Materials and Methods

### 3.1. Protein Targets

Eleven crystallographic structures of SULT1A1 available in Protein Data Bank (PDB, https://www.rcsb.org, accessed on 16 August 2021) were downloaded and analyzed. Three alloforms, SULT1A1*1, SULT1A1*2, and SULT1A1*3 were considered. The crystallographic structures with PDB ID 4GRA for SULT1A1*1 and PDB ID 1Z28 for SULT1A1*3, crystallized in their apoforms, were selected as the only representatives of these alloforms in PDB. Among the nine structures available for SULT1A1*2, three are co-crystallized with P-nitrophenol (1LS6 is co-crystallized with two molecules of P-nitrophenol), another three with 7-hydroxy-2-oxo-2H-chromene-3-carbonitrile (3QV), one with estradiol (EST), one with naphthalen-2-ol (O3V), and one is in its apoform. Based on the analysis of protein-ligand interactions in crystallographic structures, as well as the placement of loop Phe81-Ser91, which is important for the molecular clamp mechanism in SULT1A1, the PDB IDs 1LS6 co-crystallized with two molecules of P-Nitrophenol (NPO) and 2D06 co-crystallized with estradiol (EST) were selected for further consideration.

The binding site of SULT1A1 for docking was defined based on the amino acid residues, involved in the binding pockets of 1LS6 and 2D06 with their ligands.

### 3.2. Datasets Preparation

A library of natural compounds known to be ligands of SULTs was assembled. The library consists of 118 structures collected from the scientific literature [15,36,37,38,39,40,41,42,43,44,45,46,47,48,49,50,51,52,53,54,55,56,57,58,59,60,61,62,63,64] and is described in detail in the Results section. 

A library of 102 synthetic SULT1A1 ligands was created with compounds collected from Refs. [14,15,39,42,54,56,59,63,71].

A library with 1220 other natural products was taken from Ref. [72]. The 118 natural SULT ligands were not present in this library.

### 3.3. Chemical Structures Preparation and Calculation of Molecular Descriptors

In this study, the 3D structures of chemical compounds were built using MOE software v.2019.0102. The molecules were minimized with the MMFF94x force field. The molecular descriptors available in MOE were calculated (322 descriptors). They include number of atoms or chemical groups of a given type, electronic, steric, hydrophobic descriptors, shape connectivity indices, and semi-empirical quantum mechanical electronic descriptors. The semi-empirical PM3 Hamiltonian was used for calculation of the quantum mechanical descriptors. Descriptors without variance in the compound sets were removed from the subsequent analysis. 

### 3.4. Molecular Dynamics

Molecular dynamics (MD) simulations were performed on three alloforms of SULT1A1 in presence of PAP, starting from PDB IDs: 4GRA (apo), 2D06 (holo with bound EST), 1LS6 (apo), and 1Z28 (apo) to ensure a broader conformational exploration of the SULT1A1 structure for subsequent docking simulations. MD simulations were performed with NAMD 2.11 [73] with the Charmm36 force field [74]. Each system underwent a 10,000-step minimization. The system was then gradually heated from 0 K to 310 K with 1000-step/K in an NVT simulation. Then, equilibration of 2 ns was performed. Finally, a 40 ns production run was performed with an NPT simulation. Three independent MD simulations at three different initial velocity conditions were executed for each system. Finally, structural clustering was done over the conformational space generated for each of the studied systems including the binding pocket with an RMSD cutoff of 1.5 Å. Six clusters for 4GRA (the representative of SULT1A1*1), 14 clusters for 2D06 (SULT1A1*2), 10 clusters for 1LS6 (SULT1A1*2), and 9 clusters for 1Z28 (SULT1A1*3) were generated. These conformations were thoroughly analyzed and compared, focusing on the orientation of amino acid residues for the binding and the placement of structural loops responsible for the flexibility of the binding pocket part. More attention was paid to the residues Phe81 and Phe84 and the loop Phe81-Ser91, which are important for the molecular clamp mechanism in SULT1A1. Thus, four conformations—one per each of the studied systems—were finally selected for further docking.

### 3.5. Docking

Docking studies of the three groups of compounds into the active site of SULT1A1 were done, applying the docking tool in MOE. Docking was performed in protein crystallographic structures and in MD-obtained structures (as described in the previous section). The PAP co-factor was kept during docking as a part of the protein receptor. Virtual screening docking protocol was applied, the default settings were used, and the best 30 docking poses of each ligand were kept. Two scoring functions, Alpha HB and London dG, were used. London dG is the default scoring function in MOE and it accounts for the average gain/loss of rotational and translational entropy, entropy loss due to conformational flexibility, geometric imperfections of hydrogen bonds, geometric perfections of metal ligation, and desolvation energy of each atom. Alpha HB was selected as focused on compounds containing H-bond donors and acceptors. This scoring function is a linear combination of two terms: the first term estimates the steric fit of the ligand to the binding site, and the second term estimates hydrogen bond effects. 

### 3.6. Statistical Analysis

Statistical analysis, including one-way ANOVA, Principal Component Analysis (PCA), and cluster analysis, was performed with Statistica 12.7 (StatSoft. Inc., Tulsa, OK, USA, www.statistica.com/). Default settings were used in the PCA, and mean substitution was used for missing descriptor data. Cluster analysis was done with Ward’s linkage rule and squared Euclidean distance as a similarity measure. 

### 3.7. ROCS Software

ROCS software v.3.3.0.3 (OpenEye Scientific Software, Inc., Santa Fe, NM, USA https://www.eyesopen.com) was used to identify chemically similar compounds. The procedure included aligning and scoring the dataset of molecular structures of the other natural products to template molecular structures of the group of SULT ligands. The Tanimoto-Combo score (shape + “color”), which combines the shape and chemical features of the aligned datasets, was used to score the similarity. The score varies from 0 (non-similar compounds) to 2 (very similar compounds).

## 4. Conclusions

Different in silico approaches were used to analyze the chemical space of natural SULT ligands and their interactions with SULT1A1. These were compared to synthetic SULT ligands and other natural products, for which interactions with SULT have not been reported yet. 

We outlined here the natural SULT ligands diversity in their structural and physico-chemical properties. The ranges of the physico-chemical descriptors for the synthetic SULT ligands were found to be comparable or narrower than the corresponding descriptor ranges for the natural SULT ligands, but some properties in terms of polarity differed between the two groups. These differences do not impact their interactions with SULTs.

The selected descriptors of the natural SULT ligands were found to be in the lower range of descriptor values of the other natural products, except logP(o/w). The two groups of compounds were similar in relation to the number of H-bond donor atoms, logP(o/w), ASA+, and the ASA_P, but some properties differed (size, number of rings, H-bond acceptor properties, ASA−, and ASA_H), also confirmed by the grouping observed in the cluster analysis.

Combining chemical space analysis and docking of natural SULT ligands, flavonoids and similar compounds were outlined as the most favorable ligands for SULT1A1 in agreement with published data (e.g., Ref. [75]). Interestingly, the search for other natural products with similarities to the natural SULT ligands and with good predicted binding energies resulted in identification of new putative natural products interacting with SULT1A1. Further elucidation of these compounds would be necessary in order to validate them as inhibitors or substrates of SULT1A1.

## Figures and Tables

**Table 1:** Investigated groups of compounds.

Compound Group	Designation	Number of Compounds	Compound Number Range in the Paper
Natural SULT ligands (hormones, neurotransmitters, plant-derived compounds and their metabolites produced by different enzymes)	natural SULT ligands	118	1–118
SULT ligands of non-natural (synthetic) origin and their metabolites produced by different enzymes	synthetic SULT ligands	102	119–220
Other natural products for which interactions with SULTs were not reported	other natural products	1220	221–1440

**Table 2:** Statistics of the structural descriptors in the group of natural SULT ligands.

Descriptor	Mean	Minimum	Maximum	Standard Deviation
ASA+	210	99	514	66
ASA−	172	64	455	73
ASA_H	294	127	551	95
ASA_P	166	38	761	95
n_acc	4	1	24	3
n_don	3	0	17	2
rings	3	1	11	1
volume	240	107	805	106
surface	268	128	809	108
weight	272	108	1085	147
logP(o/w)	2.2	−2.1	5.3	1.2

**Table 3:** Comparison of the descriptors between the groups of natural SULT ligands (118 compounds), synthetic SULT ligands (102 compounds), and other natural products (1220 compounds) —ANOVA Fisher statistics (F) and probability values (*p*).

	Comparison of the Descriptors between the Groups of Natural and Synthetic SULT Ligands		Comparison of the Descriptors between the Groups of Natural SULT Ligands and other Natural Products	
Descriptor	F	*p*	F	*p*
ASA+	30.6	0.0000	7.0	0.0084
ASA−	0.0	0.8895	13.5	0.0002
ASA_H	13.1	0.0004	91.9	0.0000
ASA_P	37.7	0.0000	7.8	0.0052
n_acc	33.8	0.0000	18.4	0.0000
n_don	22.1	0.0000	2.1	0.1501
rings	6.9	0.0093	28.6	0.0000
volume	1.1	0.2860	81.0	0.0000
surface	1.1	0.2949	77.6	0.0000
weight	3.2	0.0746	57.1	0.0000
logP(o/w)	31.8	0.0000	0.7	0.4101

## Data Availability

The in-house library of SULTs ligands is freely available at http://biomed.bas.bg/qsarmm/.

## References

[1] Sun H., Scott D.O. (2010). Structure-Based Drug Metabolism Predictions for Drug Design. Chem. Biol. Drug Des..

[2] Testa B., Pedretti A., Vistoli G. (2012). Reactions and Enzymes in the Metabolism of Drugs and Other Xenobiotics. Drug Discov. Today.

[3] Shimada T. (2006). Xenobiotic-Metabolizing Enzymes Involved in Activation and Detoxification of Carcinogenic Polycyclic Aromatic Hydrocarbons. Drug Metab. Pharmacokinet..

[4] Moroy G., Martiny V.Y., Vayer P., Villoutreix B.O., Miteva M.A. (2012). Toward in Silico Structure-Based ADMET Prediction in Drug Discovery. Drug Discov. Today.

[5] Pratt W.B., Taylor P. (1990). Principles of Drug Action: The Basis of Pharmacology.

[6] Tibbs Z.E., Rohn-Glowacki K.J., Crittenden F., Guidry A.L., Falany C.N. (2015). Structural Plasticity in the Human Cytosolic Sulfotransferase Dimer and Its Role in Substrate Selectivity and Catalysis. Drug Metab. Pharmacokinet..

[7] Dong D., Ako R., Wu B. (2012). Crystal Structures of Human Sulfotransferases: Insights into the Mechanisms of Action and Substrate Selectivity. Expert Opin. Drug Metab. Toxicol..

[8] Gamage N., Barnett A., Hempel N., Duggleby R.G., Windmill K.F., Martin J.L., McManus M.E. (2006). Human Sulfotransferases and Their Role in Chemical Metabolism. Toxicol. Sci..

[9] Bojarová P., Williams S.J. (2008). Sulfotransferases, Sulfatases and Formylglycine-Generating Enzymes: A Sulfation Fascination. Curr. Opin. Chem. Biol..

[10] Chapman E., Best M.D., Hanson S.R., Wong C.-H. (2004). Sulfotransferases: Structure, Mechanism, Biological Activity, Inhibition, and Synthetic Utility. Angew. Chem. Int. Ed..

[11] Coughtrie M.W.H. (2016). Function and Organization of the Human Cytosolic Sulfotransferase (SULT) Family. Chem. Biol. Interact..

[12] Vrba J., Papoušková B., Kosina P., Lněničková K., Valentová K., Ulrichová J. (2020). Identification of Human Sulfotransferases Active towards Silymarin Flavonolignans and Taxifolin. Metabolites.

[13] Brand W., Boersma M.G., Bik H., den Hil E.F.H., Vervoort J., Barron D., Meinl W., Glatt H., Williamson G., van Bladeren P.J. (2010). Phase II Metabolism of Hesperetin by Individual UDP-Glucuronosyltransferases and Sulfotransferases and Rat and Human Tissue Samples. Drug Metab. Dispos..

[14] Martiny V.Y., Carbonell P., Lagorce D., Villoutreix B.O., Moroy G., Miteva M.A. (2013). In Silico Mechanistic Profiling to Probe Small Molecule Binding to Sulfotransferases. PLoS ONE.

[15] Allali-Hassani A., Pan P.W., Dombrovski L., Najmanovich R., Tempel W., Dong A., Loppnau P., Martin F., Thonton J., Edwards A.M. (2007). Structural and Chemical Profiling of the Human Cytosolic Sulfotransferases. PLoS Biol..

[16] Dajani R., Hood A.M., Coughtrie M.W.H. (1998). A Single Amino Acid, Glu146, Governs the Substrate Specificity of a Human Dopamine Sulfotransferase, SULT1A3. Mol. Pharmacol..

[17] Lee K.A., Fuda H., Lee Y.C., Negishi M., Strott C.A., Pedersen L.C. (2003). Crystal Structure of Human Cholesterol Sulfotransferase (SULT2B1b) in the Presence of Pregnenolone and 3′-Phosphoadenosine 5′-Phosphate: Rationale for Specificity Differences between Prototypical SULT2A1 and the SULT2B1 Isoforms. J. Biol. Chem..

[18] Wang T., Cook I., Leyh T.S. (2017). The NSAID Allosteric Site of Human Cytosolic Sulfotransferases. J. Biol. Chem..

[19] Cook I., Wang T., Girvin M., Leyh T.S. (2016). The Structure of the Catechin-Binding Site of Human Sulfotransferase 1A1. Proc. Natl. Acad. Sci. USA.

[20] Cook I., Wang T., Falany C.N., Leyh T.S. (2015). The Allosteric Binding Sites of Sulfotransferase 1A1. Drug Metab. Dispos..

[21] Cook I., Wang T., Falany C.N., Leyh T.S. (2013). High Accuracy in Silico Sulfotransferase Models. J. Biol. Chem..

[22] Cook I., Wang T., Almo S.C., Kim J., Falany C.N., Leyh T.S. (2013). The Gate That Governs Sulfotransferase Selectivity. Biochemistry.

[23] Zhu J., Qi R., Liu Y., Zhao L., Han W. (2019). Mechanistic Insights into the Effect of Ligands on Structural Stability and Selectivity of Sulfotransferase 2A1 (SULT2A1). ACS Omega.

[24] Rakers C., Schumacher F., Meinl W., Glatt H., Kleuser B., Wolber G. (2016). In Silico Prediction of Human Sulfotransferase 1E1 Activity Guided by Pharmacophores from Molecular Dynamics Simulations. J. Biol. Chem..

[25] Dudas B., Toth D., Perahia D., Nicot A.B., Balog E., Miteva M.A. (2021). Insights into the Substrate Binding Mechanism of SULT1A1 through Molecular Dynamics with Excited Normal Modes Simulations. Sci. Rep..

[26] Gamage N.U., Duggleby R.G., Barnett A.C., Tresillian M., Latham C.F., Liyou N.E., McManus M.E., Martin J.L. (2003). Structure of a Human Carcinogen-Converting Enzyme, SULT1A1: Structural and Kinetic Implications of Substrate Inhibition. J. Biol. Chem..

[27] Hempel N., Gamage N., Martin J.L., McManus M.E. (2007). Human Cytosolic Sulfotransferase SULT1A1. Int. J. Biochem. Cell Biol..

[28] Cisneros K.V., Agarwal V., James M.O. (2019). Sulfonation and Glucuronidation of Hydroxylated Bromodiphenyl Ethers in Human Liver. Chemosphere.

[29] Zeng M., Yang L., He D., Li Y., Shi M., Zhang J. (2017). Metabolic Pathways and Pharmacokinetics of Natural Medicines with Low Permeability. Drug Metab. Rev..

[30] Wu B., Basu S., Meng S., Wang X., Zhang S. (2011). Regioselective Sulfation and Glucuronidation of Phenolics: Insights into the Structural Basis of Conjugation. Curr. Drug Metab..

[31] Glatt H. (2000). Sulfotransferases in the Bioactivation of Xenobiotics. Chem. Biol. Interact..

[32] Vrba J., Papoušková B., Lněničková K., Kosina P., Křen V., Ulrichová J. (2020). Identification of UDP-Glucuronosyltransferases Involved in the Metabolism of Silymarin Flavonolignans. J. Pharm. Biomed. Anal..

[33] Vrba J., Papoušková B., Roubalová L., Zatloukalová M., Biedermann D., Křen V., Valentová K., Ulrichová J., Vacek J. (2018). Metabolism of Flavonolignans in Human Hepatocytes. J. Pharm. Biomed. Anal..

[34] Vacek J., Papoušková B., Kosina P., Vrba J., Křen V., Ulrichová J. (2012). Biotransformation of Flavonols and Taxifolin in Hepatocyte in Vitro Systems as Determined by Liquid Chromatography with Various Stationary Phases and Electrospray Ionization-Quadrupole Time-of-Flight Mass Spectrometry. J. Chromatogr. B.

[35] Vrba J., Kren V., Vacek J., Papouskova B., Ulrichova J. (2012). Quercetin, Quercetin Glycosides and Taxifolin Differ in Their Ability to Induce AhR Activation and CYP1A1 Expression in HepG2 Cells. Phytother. Res..

[36] Falany C.N., Wheeler J., Oh T.S., Falany J.L. (1994). Steroid Sulfation by Expressed Human Cytosolic Sulfotransferases. J. Steroid Biochem. Mol. Biol..

[37] Eaton E.A., Walle U.K., Lewis A.J., Hudson T., Wilson A.A., Walle T. (1996). Flavonoids, Potent Inhibitors of the Human P-Form Phenolsulfotransferase. Potential Role in Drug Metabolism and Chemoprevention. Drug Metab. Dispos..

[38] Harris R.M., Hawker R.J., Langman M.J.S., Singh S., Waring R.H. (1998). Inhibition of Phenolsulphotransferase by Salicylic Acid: A Possible Mechanism by Which Aspirin May Reduce Carcinogenesis. Gut.

[39] Dajani R., Cleasby A., Neu M., Wonacott A.J., Jhoti H., Hood A.M., Modi S., Hersey A., Taskinen J., Cooke R.M. (1999). X-Ray Crystal Structure of Human Dopamine Sulfotransferase, SULT1A3: Molecular Modeling and Quantitative Structure-Activity Relationship Analysis Demonstrate a Molecu-Lar Basis for Sulfotransferase Substrate Specificity. J. Biol. Chem..

[40] Harris R.M., Waring R.H., Kirk C.J., Hughes P.J. (2000). Sulfation of “Estrogenic” Alkylphenols and 17β-Estradiol by Human Platelet Phenol Sulfotransferases. J. Biol. Chem..

[41] Spink B.C., Katz B.H., Hussain M.M., Pang S., Connor S.P., Aldous K.M., Gierthy J.F., Spink D.C. (2000). SULT1A1 Catalyzes 2-Methoxyestradiol Sulfonation in MCF-7 Breast Cancer Cells. Carcinogenesis.

[42] Glatt H., Boeing H., Engelke C.E.H., Ma L., Kuhlow A., Pabel U., Pomplun D., Teubner W., Meinl W. (2001). Human Cytosolic Sulphotransferases: Genetics, Characteristics, Toxicological Aspects. Mutat. Res. Mol. Mech. Mutagen..

[43] Honma W., Kamiyama Y., Yoshinari K., Sasano H., Shimada M., Nagata K., Yamazoe Y. (2001). Enzymatic Characterization and Interspecies Difference of Phenol Sulfotransferases, ST1A Forms. Drug Metab. Dispos..

[44] Li X., Clemens D.L., Cole J.R., Anderson R.J. (2001). Characterization of Human Liver Thermostable Phenol Sulfotransferase (SULT1A1) Allozymes with 3,3′,5-Triiodothyronine as the Substrate. J. Endocrinol..

[45] Mesía-Vela S., Sańchez R.I., Estrada-Muñiz E., Alavez-Solano D., Torres-Sosa C., Jiménez-Estrada M., Reyes-Chilpa R., Kauffman F.C. (2001). Natural Products Isolated from Mexican Medicinal Plants: Novel Inhibitors of Sulfotransferases, SULT1A1 and SULT2A1. Phytomedicine.

[46] Mesía-Vela S., Kauffman F.C. (2003). Inhibition of Rat Liver Sulfotransferases SULT1A1 and SULT2A1 and Glucuronosyltransferase by Dietary Flavonoids. Xenobiotica.

[47] Taskinen J., Ethell B.T., Pihlavisto P., Hood A.M., Burchell B., Coughtrie M.W.H. (2003). Conjugation of Catechols by Recombinant Human Sulfotransferases, Udp-Glucuronosyltransferases, and Soluble Catechol O-Methyltransferase: Structure-Conjugation Relationships and Predictive Models. Drug Metab. Dispos..

[48] Yeh C.-T., Yen G.-C. (2003). Effects of Phenolic Acids on Human Phenolsulfotransferases in Relation to Their Antioxidant Activity. J. Agric. Food Chem..

[49] Harris R.M., Wood D.M., Bottomley L., Blagg S., Owen K., Hughes P.J., Waring R.H., Kirk C.J. (2004). Phytoestrogens Are Potent Inhibitors of Estrogen Sulfation: Implications for Breast Cancer Risk and Treatment. J. Clin. Endocrinol. Metab..

[50] Nakano H., Ogura K., Takahashi E., Harada T., Nishiyama T., Muro K., Hiratsuka A., Kadota S., Watabe T. (2004). Regioselective Monosulfation and Disulfation of the Phytoestrogens Daidzein and Genistein by Human Liver Sulfotransferases. Drug Metab. Pharmacokinet..

[51] Aust S., Jaeger W., Klimpfinger M., Mayer K., Baravalle G., Ekmekcioglu C., Thalhammer T. (2005). Biotransformation of Melatonin in Human Breast Cancer Cell Lines: Role of Sulfotransferase 1A1. J. Pineal Res..

[52] Meinl W., Pabel U., Osterloh-Quiroz M., Hengstler J.G., Glatt H. (2006). Human Sulphotransferases Are Involved in the Activation of Aristolochic Acids and Are Expressed in Renal Target Tissue. Int. J. Cancer.

[53] Nishimuta H., Ohtani H., Tsujimoto M., Ogura K., Hiratsuka A., Sawada Y. (2007). Inhibitory Effects of Various Beverages on Human Recombinant Sulfotransferase Isoforms SULT1A1 and SULT1A3. Biopharm. Drug Dispos..

[54] Riches Z., Bloomer J.C., Coughtrie M.W.H. (2007). Comparison of 2-Aminophenol and 4-Nitrophenol as in Vitro Probe Substrates for the Major Human Hepatic Sulfotransferase, SULT1A1, Demonstrates Improved Selectivity with 2-Aminophenol. Biochem. Pharmacol..

[55] Ung D., Nagar S. (2007). Variable Sulfation of Dietary Polyphenols by Recombinant Human Sulfotransferase (SULT) 1A1 Genetic Variants and SULT1E1. Drug Metab. Dispos..

[56] Yasuda S., Idell S., Fu J., Carter G., Snow R., Liu M.-C. (2007). Cigarette Smoke Toxicants as Substrates and Inhibitors for Human Cytosolic SULTs. Toxicol. Appl. Pharmacol..

[57] Yasuda S., Liu M.-Y., Suiko M., Sakakibara Y., Liu M.-C. (2007). Hydroxylated Serotonin and Dopamine as Substrates and Inhibitors for Human Cytosolic SULT1A3. J. Neurochem..

[58] Saruwatari A., Okamura S., Nakajima Y., Narukawa Y., Takeda T., Tamura H. (2008). Pomegranate Juice Inhibits Sulfoconjugation in Caco-2 Human Colon Carcinoma Cells. J. Med. Food.

[59] Waring R.H., Ayers S., Gescher A.J., Glatt H.-R., Meinl W., Jarratt P., Kirk C.J., Pettitt T., Rea D., Harris R.M. (2008). Phytoestrogens and Xenoestrogens: The Contribution of Diet and Environment to Endocrine Disruption. J. Steroid Biochem. Mol. Biol..

[60] Huang C., Chen Y., Zhou T., Chen G. (2009). Sulfation of Dietary Flavonoids by Human Sulfotransferases. Xenobiotica.

[61] Senggunprai L., Yoshinari K., Yamazoe Y. (2009). Inhibitory Effects of Kynurenic Acid, a Tryptophan Metabolite, and Its Derivatives on Cytosolic Sulfotransferases. Biochem. J..

[62] Wong C.C., Meinl W., Glatt H.-R., Barron D., Stalmach A., Steiling H., Crozier A., Williamson G. (2010). In Vitro and in Vivo Conjugation of Dietary Hydroxycinnamic Acids by UDP-Glucuronosyltransferases and Sulfotransferases in Humans. J. Nutr. Biochem..

[63] Kurogi K., Chepak A., Hanrahan M.T., Liu M.-Y., Sakakibara Y., Suiko M., Liu M.-C. (2014). Sulfation of Opioid Drugs by Human Cytosolic Sulfotransferases: Metabolic Labeling Study and Enzymatic Analysis. Eur. J. Pharm. Sci..

[64] Han Z., Xi Y., Luo L., Zhou C., Kurogi K., Sakakibara Y., Suiko M., Liu M.-C. (2016). Sulfate Conjugation of Daphnetin by the Human Cytosolic Sulfotransferases. J. Ethnopharmacol..

[65] Sneath P.H.A., Sokal R.R. (1973). Numerical Taxonomy: The Principles and Practice of Numerical Classification.

[66] Glatt H., Meinl W. (2004). Pharmacogenetics of Soluble Sulfotransferases (SULTs). Naunyn. Schmiedebergs Arch. Pharmacol..

[67] Whittemore R.M., Pearce L.B., Roth J.A. (1986). Purification and Kinetic Characterization of a Phenol-Sulfating Form of Phenol Sulfotransferase from Human Brain. Arch. Biochem. Biophys..

[68] Costa M.G.S., Batista P.R., Bisch P.M., Perahia D. (2015). Exploring Free Energy Landscapes of Large Conformational Changes: Molecular Dynamics with Excited Normal Modes. J. Chem. Theory Comput..

[69] Zhang X., Song J., Shi X., Miao S., Li Y., Wen A. (2013). Absorption and Metabolism Characteristics of Rutin in Caco-2 Cells. Sci. World, J..

[70] Chávez-Hernández A.L., Sánchez-Cruz N., Medina-Franco J.L. (2020). A Fragment Library of Natural Products and Its Comparative Chemoinformatic Characterization. Mol. Inform..

[71] Squirewell E.J., Duffel M.W. (2015). The Effects of Endoxifen and Other Major Metabolites of Tamoxifen on the Sulfation of Estradiol Catalyzed by Human Cytosolic Sulfotransferases HSULT1E1 and HSULT1A1*1. Drug Metab. Dispos..

[72] Lagarde N., Rey J., Gyulkhandanyan A., Tufféry P., Miteva M.A., Villoutreix B.O. (2018). Online Structure-Based Screening of Purchasable Approved Drugs and Natural Compounds: Retrospective Examples of Drug Repositioning on Cancer Targets. Oncotarget.

[73] Phillips J.C., Braun R., Wang W., Gumbart J., Tajkhorshid E., Villa E., Chipot C., Skeel R.D., Kalé L., Schulten K. (2005). Scalable Molecular Dynamics with NAMD. J. Comput. Chem..

[74] Best R.B., Zhu X., Shim J., Lopes P.E.M., Mittal J., Feig M., MacKerell A.D. (2012). Optimization of the Additive CHARMM All-Atom Protein Force Field Targeting Improved Sampling of the Backbone ϕ, ψ and Side-Chain Χ1 and Χ2 Dihedral Angles. J. Chem. Theory Comput..

[75] James M.O., Ambadapadi S. (2013). Interactions of Cytosolic Sulfotransferases with Xenobiotics. Drug Metab. Rev..

